# Jasmonic Acid Methyl Ester Induces Xylogenesis and Modulates Auxin-Induced Xylary Cell Identity with NO Involvement

**DOI:** 10.3390/ijms20184469

**Published:** 2019-09-10

**Authors:** Federica Della Rovere, Laura Fattorini, Marilena Ronzan, Giuseppina Falasca, Maria Maddalena Altamura, Camilla Betti

**Affiliations:** 1 Department of Environmental Biology, Sapienza University of Rome, Piazzale Aldo Moro 5, 00185 Rome, Italy; 2 Department of Medicine, University of Perugia, Piazzale Menghini 8/9, 06132 Perugia, Italy

**Keywords:** adventitious rooting, auxin, ectopic metaxylem, ectopic protoxylem, ethylene, hypocotyl, jasmonates, nitric oxide, xylogenesis

## Abstract

In *Arabidopsis* basal hypocotyls of dark-grown seedlings, xylary cells may form from the pericycle as an alternative to adventitious roots. Several hormones may induce xylogenesis, as Jasmonic acid (JA), as well as indole-3-acetic acid (IAA) and indole-3-butyric acid (IBA) auxins, which also affect xylary identity. Studies with the ethylene (ET)-perception mutant *ein3eil1* and the ET-precursor 1-aminocyclopropane-1-carboxylic acid (ACC), also demonstrate ET involvement in IBA-induced ectopic metaxylem. Moreover, nitric oxide (NO), produced after IBA/IAA-treatments, may affect JA signalling and interact positively/negatively with ET. To date, NO-involvement in ET/JA-mediated xylogenesis has never been investigated. To study this, and unravel JA-effects on xylary identity, xylogenesis was investigated in hypocotyls of seedlings treated with JA methyl-ester (JAMe) with/without ACC, IBA, IAA. Wild-type (wt) and *ein3eil1* responses to hormonal treatments were compared, and the NO signal was quantified and its role evaluated by using NO-donors/scavengers. Ectopic-protoxylem increased in the wt only after treatment with JAMe(10 μM), whereas in *ein3eil1* with any JAMe concentration. NO was detected in cells leading to either xylogenesis or adventitious rooting, and increased after treatment with JAMe(10 μM) combined or not with IBA(10 μM). Xylary identity changed when JAMe was applied with each auxin. Altogether, the results show that xylogenesis is induced by JA and NO positively regulates this process. In addition, NO also negatively interacts with ET-signalling and modulates auxin-induced xylary identity.

## 1. Introduction

In vascular plants, the process leading to the differentiation of the xylem conducting elements (xylary elements, XEs) is still not fully understood. The XEs differentiate either from the procambium, and are named protoxylem and metaxylem, or from the vascular cambium (deuteroxylem). However, XEs may be produced also by other cell types, both in planta and, mainly, in vitro. Auxin is necessary for XE induction and early development [1], acting through the expression of the same genes both in planta and in vitro [2]. In in vitro cultured systems, XE formation occurs either via a direct trans-differentiation event, like in zinnia (*Zinnia elegans*) mesophyll cells [3], or via a cell de-differentiation process, followed by mitotic proliferation, as in *Arabidopsis thaliana* stem thin cell layers (TCLs) [4]. Additionally, other cell types than those of the procambium/cambium may be triggered in planta to either trans-differentiate into XEs, e.g., after a mechanical wounding [3], or to proliferate. The latter process causes the formation of cells that only subsequently differentiate into supernumerary XEs and has been observed in the hypocotyls of *Arabidopsis* dark-grown seedlings treated with naphthaleneacetic acid (NAA) [5]. The xylogenic response in planta, defined as ectopic xylary cell formation, occurs because vascular pattern formation is not cell clonal. XE-arrangement can be, in fact, altered by local signals, e.g., derived by phytohormones, or modified in response to environmental stimuli [6]. Xylogenesis is always preceded by an endogenous auxin (indole-3-acetic acid, IAA) accumulation [7,8,9]. In the basal hypocotyl of *Arabidopsis* dark-grown seedlings, ectopic XEs formation in the pericycle periclinal derivatives is controlled by either IAA, or indole-3-butyric acid (IBA), one of the possible IAA precursor ([10,11] and references therein), or the synthetic auxin NAA [5,7]. Moreover, in lettuce, pith cells trans-differentiate into XEs in the presence of NAA and either the ethylene (ET) precursor 1-aminocyclopropane-1-carboxylic acid (ACC), or the ET-releasing compound ethephon, thus demonstrating that ET and auxin are both required for xylogenesis in this species [12]. Xylem cells differentiation requires the realization of programmed cell death (PCD) [3]; it has been shown that ET is involved in PCD signalling during early and late stages of XE differentiation, at least in zinnia cell cultures [13,14]. Furthermore, exogenous ACC/ET stimulates xylem formation in stems/cuttings of gymnosperms and angiosperms [15,16,17]. In *Arabidopsis* dark-grown seedlings, it has been recently demonstrated that ET is involved in ectopic xylem formation through the activation of the ETHYLENE INSENSITIVE 3 (EIN3) and EIN3-LIKE 1 (EIL1) transcription factors (TFs) [11]. Both these TFs activate numerous ET-responsive genes [18] and when mutated cause complete ET-insensitivity ([19] and references therein) and persistent auxin-sensitivity [20]. In the basal hypocotyl of *Arabidopsis* dark-grown seedlings, the treatment with IAA (10 μM) induces exclusively ectopic protoxylem formation, whereas exogenous IBA (10 μM) only ectopic metaxylem, showing that the two auxins have different effects on XE identity. Both ectopic protoxylem and metaxylem are induced by ACC, when applied to the seedlings at 0.1 μM concentration together with IAA (10 μM), but only ectopic metaxylem is formed when seedlings are treated with ACC in combination with IBA (10 μM) [11].

It has been reported that both antagonistic and synergistic interactions occur also between ET and jasmonates (JAs) in numerous morphogenic and defense responses, mainly relying on EIN3/EIL1 functions [21].

In zinnia cell cultures genes involved in jasmonic acid (JA) synthesis and signalling are expressed at early stages of the xylogenic process [1]. In *Arabidopsis*, JAs trigger cambium cell division leading to deuteroxylem formation in shoots [22]. In addition, the early release of JA, after treatment with JA methyl ester (JAMe), and due to its demethylation [23], induces extra xylem in roots [24] modulating XE formation by controlling polar auxin transport in the vascular tissues [25]. However, the effect of JA seems to be dose-dependent, because low xylogenesis occurs after treatment with 1 μM JAMe, while high xylogenesis with 10 μM JAMe [24]. Moreover, when JAMe is applied at 10 µM concentration, xylogenesis is stimulated also in *Arabidopsis* TCLs cultured with IBA and Kinetin [26].

Nitric oxide (NO) is a reactive free radical molecule, which serves as a messenger in several cell differentiation events [27], including PCD [28]. In *Zinnia elegans*, a NO burst occurs before secondary cell wall formation and cell autolysis both in the differentiating xylem of the vascular bundles and in mesophyll cells undergoing trans-differentiation into XEs ([29] and references therein). The production of NO might be stimulated by auxin and be also spatially and temporally controlled ([27] and references therein). Moreover, NO can also affect organ elongation by regulating auxin transport, as seen in rice roots [30].

Exogenous NO may negatively impact ET synthesis; however, it may also stimulate ET production ([27] and references therein), and affect ET-signalling at the level of ETHYLENE INSENSITIVE 2 (EIN2) [31]. In the presence of ET, EIN2 activates a TF cascade, involving EIN3 and EIL1, which leads to specific plant developmental responses [32]. However, a possible interaction between NO and ET-signalling mediated by EIN3/EIL1 has been never demonstrated in the process of xylogenesis. Recent studies show that ET and NO may act as transducers of PCD signalling in early stages of xylogenesis, and as co-actors at later stages of cell death [33,34]. In addition, ET and NO are both involved in adventitious rooting in competition with xylogenesis in numerous plant species, including *Arabidopsis* ([11,35] and references therein).

A crosstalk between NO and JA may occur in some developmental processes possibly mediated by *OPR3*, a gene coding for OPDA-reductase 3 (OPR3), which converts 12-oxophytodienoic acid (OPDA) into the first precursor of JA ([26] and other references therein). In *Arabidopsis*, *OPR3* expression is increased by NO, resulting into an increased JA production ([36] and references therein), although an OPR3-independent JA biosynthetic pathway has also been recently discovered in this species [37]. Moreover, in *Taxus* cells, exogenous JAMe is known to stimulate NO synthase to induce NO production [38]. Therefore, it seems that JA and NO can modulate each other production, but their possible interaction in xylogenesis is not yet characterized.

The aim of the present research is to determine the involvement of JA, ET and NO in the control of endogenous/exogenous-auxin-induced xylogenesis through a possible crosstalk mediated by EIN3/EIL1. To this aim, ectopic xylem formation was investigated in the hypocotyl of dark-grown *Arabidopsis* seedlings exposed to various concentrations of JAMe with/without ACC, and exogenous IBA or IAA. The xylogenic response in the wild-type (wt) was compared with the *ein3eil1* mutant, the NO production monitored, and the NO putative role evaluated by measuring the effects of treatments with NO-donors/scavengers.

Results show that the ectopic formation of protoxylem was enhanced by JAMe when applied alone at a specific concentration (10 μM). The *ein3eil1* mutant was sensitive to JAMe. In fact, the stimulation of XEs mediated by JAMe occurred also in the mutant, suggesting that a negative interaction between JA and ET-signalling is involved in xylogenesis. This was strengthened by the reduction in xylogenesis observed in the wt after the combined application of JAMe with the ET-precursor ACC, in comparison with JAMe single treatments. Nitric oxide was detected at early stages of both xylogenesis and adventitious rooting in the hypocotyl pericycle cells, and its production was highly enhanced by JAMe (10 μM).

Surprisingly, xylogenesis did not increase quantitatively with any JAMe treatment combined with either IBA or IAA. Conversely, the xylary identity changed, in comparison with either auxin single treatments. In addition, the IBA/IAA-induced adventitious rooting was increased by the same JAMe concentration enhancing xylogenesis when applied alone. This suggests a role for JA in modulating adventitious rooting and xylogenesis programs in the same target cells through an interaction with NO.

The role of JA in inducing xylogenesis and in modifying xylary cell identity is further discussed.

## 2. Results

### 2.1. A Specific Concentration of Exogenous JAMe Enhances Ectopic Protoxylem Formation but its Combination with ACC Reduces it, without Affecting Xylary Identity

**Figure 1:**
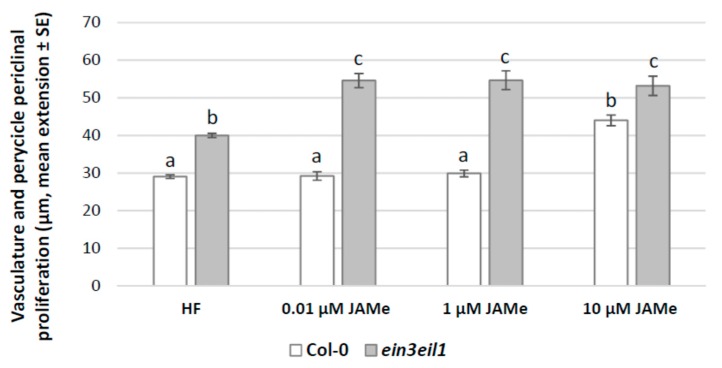
Pericycle periclinal proliferation in the basal hypocotyl of *Arabidopsis* Col-0 (wt) and *ein3eil1* double mutant seedlings either cultured with different JAMe concentrations (0.01, 1, 10 μM) or in hormone-free (HF) condition. Mean radial extension (± Standard error (SE)) of the vasculature and the pericycle periclinal proliferation in the basal 5 mm of the hypocotyl. Different letters among different treatments within the same genotype, or between the two genotypes within the same treatment, indicate significant differences at least at *p* < 0.001. The same letters indicate no significant difference. Two-way ANOVA followed by Tukey’s post-test. *n* = 30.

**Figure 2:**
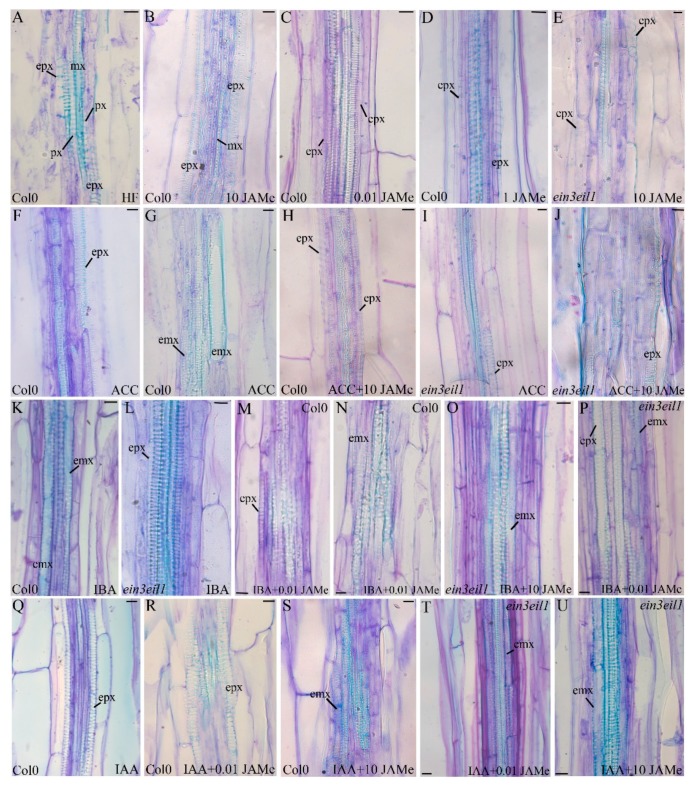
Xylogenesis in the basal hypocotyl of *Arabidopsis* Col-0 (wt) and *ein3eil1* double mutant seedlings dark-grown for 22 DAS with different treatments. (**A**) Ectopic protoxylem elements occasionally formed by periclinal pericycle cell divisions in the basal 5 mm of Col-0 hypocotyl in the absence of exogenous hormones (hormone-free, HF). (**B**–**U**) Ectopic protoxylem or ectopic metaxylem elements formed in Col-0 (**B**–**D**,**F**–**H**,**K**,**M**,**N**,**Q**–**S**) or *ein3eil1* (**E**,**I**,**J**,**L**,**O**,**P**,**T**,**U**) basal hypocotyl treated with: 0.01 µM JAMe (**C**), 1 µM JAMe (**D**), 10 µM JAMe (**B**,**E**), 0.1 µM ACC (**F**,**G**,**I**), 0.1 µM ACC + 10 µM JAMe (**H**,**J**), 10 µM IBA (**K**,**L**), 10 µM IBA + 0.01 µM JAMe (**M**,**N**,**P**), 10 µM IBA + 10 µM JAMe (**O**), 10 µM IAA (**Q**), 10 µM IAA + 0.01 µM JAMe (**R**,**T**), 10 µM IAA + 10 µM JAMe (**S**,**U**). emx, ectopic metaxylem; epx, ectopic protoxylem; mx, metaxylem; px, protoxylem. Radial longitudinal sections stained with toluidine blue. Scale bars = 10 µm (**B**–**U**), 20 µm (**A**).

The hypocotyl base of seedlings grown in hormone free (HF) condition showed a sporadic cell proliferation in the pericycle (Figure 1), with occasional ectopic protoxylem formation (Figure 2A, and Table 1). Pericycle proliferation did not increase with 0.01 μM and 1 μM JAMe, but a significant increase was observed with 10 μM JAMe (Figure 1). Independently from the treatment, the proliferation was always associated with ectopic xylary cells (XEs) formation (Table 1).

In the mutant seedlings, the cell proliferation with XEs from the pericycle significantly increased compared to the wt with any JAMe treatment condition (Figure 1). Thus, in contrast with the JA-insensitivity observed in other morphogenic processes, e.g., root hair formation [39], the *ein3eil1* mutant perceived JA, giving rise to a pericycle xylogenic proliferation with significant increases even at the lowest JAMe dose tested. Independently from treatment and genotype, only ectopic protoxylem was formed (Figure 2B–E and Table 1). This showed that the JA deriving from the exogenous JAMe did not change the xylary cell identity determined by endogenous hormones, even in the impaired ET-perception mutant.

**Figure 3:**
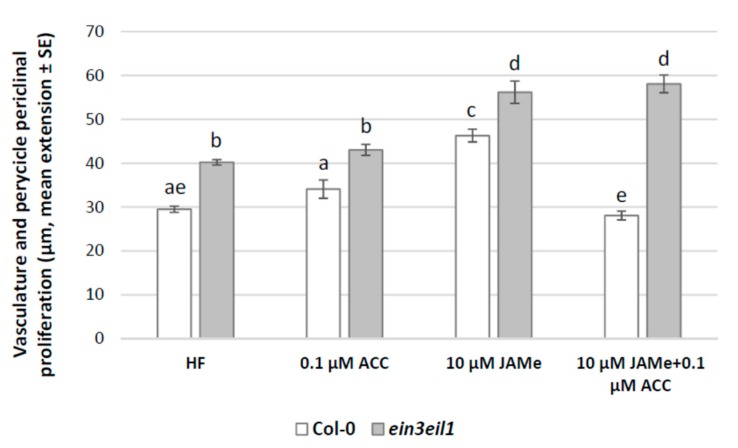
Pericycle periclinal proliferation in the basal hypocotyl of *Arabidopsis* Col-0 (wt) and *ein3eil1* double mutant seedlings either cultured with ACC (0.1μM), or with JAMe (10 μM) alone or combined with ACC (0.1μM), and without exogenous hormones (hormone-free, HF). Mean radial extension (± Standard Error (SE)) of the vasculature plus pericycle periclinal proliferation in the basal 5 mm of the hypocotyl. Different letters among different treatments within the same genotype, or between the two genotypes within the same treatment, indicate significant differences at least at *p* < 0.05. The same letters indicate no significant difference. Two-way ANOVA followed by Tukey’s post-test. *n* = 30.

In the basal portion of wt seedlings treated with ACC a pericycle periclinal proliferation with xylogenesis comparable to HF condition was observed (Figure 3). However, both protoxylem and metaxylem elements were formed (Figure 2F,G, and Table 1). The extension of the vasculature, including the portion with pericycle proliferation, and the number of XEs formed were significantly reduced after ACC treatment in comparison with 10 μM JAMe (Figure 3 and Table 1). Moreover, there was also a significant decrease in proliferation with xylogenesis when the two compounds were combined in comparison with both ACC or JAMe alone (Figure 3). Interestingly, only protoxylem was formed in the combined treatment, as with JAMe (Figure 2B,H, in comparison, and Table 1).

In the *ein3eil1* mutant, the periclinal proliferation with xylogenesis observed in the presence of ACC was comparable to that obtained in HF condition (Figure 3), in accordance with the mutant insensitivity to the ACC-derived ET. However, the xylogenic proliferation observed in the ACC and JAMe combined treatment was higher than the wt, reaching values not significantly different from those of JAMe alone (Figure 3). This confirmed the mutant responsiveness to JAMe and the stimulating effect of the latter on xylogenesis (Figure 1 and Figure 3, and Table 1). In addition, only ectopic protoxylem was formed by the mutant (Figure 2I,J and Table 1).

Taken together, the results supported the hypothesis that JA positive role on xylogenesis was negatively affected by ET perception, with no effect on JAMe-determined xylary cell identity.

### 2.2. JAMe Changes the Xylary Cell Identity Determined by Exogenous IBA or IAA and Promotes Adventitious Rooting when Applied with IAA/IBA

Experiments were carried out to identify possible roles of JAMe on xylary cell identity in cooperation/antagonism with either IAA or IBA control, and in the presence/absence of ET perception through EIN3 and EIL1.

**Figure 4:**
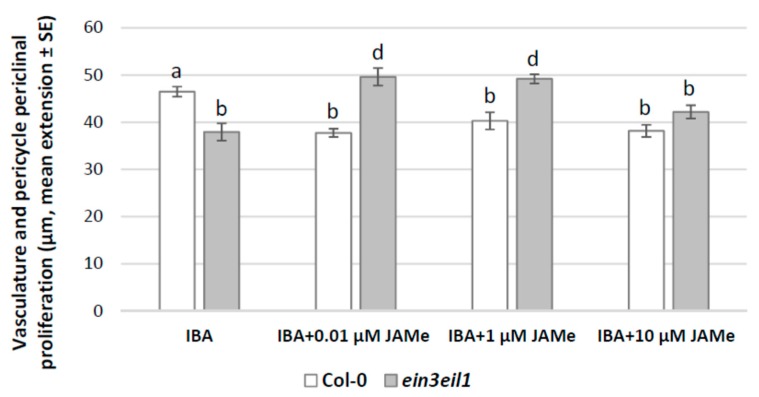
Pericycle periclinal proliferation in the basal hypocotyl of *Arabidopsis* Col-0 (wt) and *ein3eil1* double mutant seedlings either cultured with IBA (10 μM) or with IBA combined with 0.01, 1 or 10 μM JAMe. Mean radial extension (± standard error (SE)) of the vasculature plus pericycle periclinal proliferation in the basal 5 mm of the hypocotyls. Different letters among different treatments within the same genotype, or between the two genotypes within the same treatment, indicate significant differences at least at *p* < 0.05. The same letters indicate no significant difference. Two-way ANOVA followed by Tukey’s post-test. *n* = 30.

**Figure 5:**
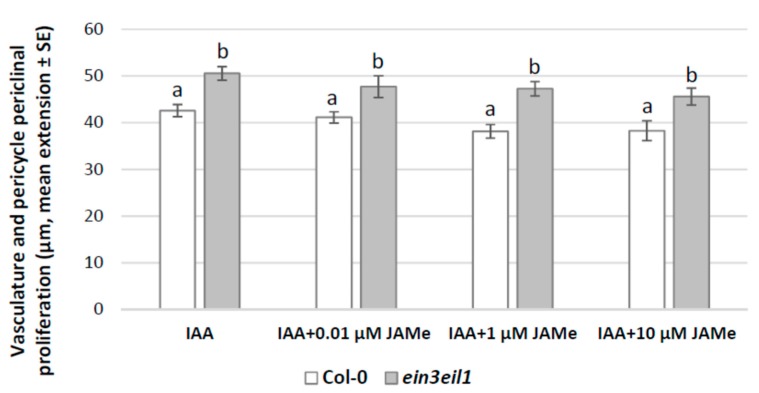
Pericycle periclinal proliferation in the basal hypocotyl of *Arabidopsis* Col-0 (wt) and *ein3eil1* double mutant seedlings either cultured with IAA (10 μM) or with IAA combined with 0.01, 1 or 10 μM JAMe. Mean radial extension (± standard error (SE)) of the vasculature plus pericycle periclinal proliferation in the basal 5 mm of the hypocotyl. Different letters among different treatments within the same genotype, or between the two genotypes within the same treatment, indicate significant differences at least at *p* < 0.05. The same letters indicate no significant difference. Two-way ANOVA followed by Tukey’s post-test. *n* = 30.

The pericycle periclinal proliferation with xylogenesis and the number of XEs formed (Table 1) in the wt basal hypocotyl were comparable after IBA, IAA or 10 μM JAMe treatments, and noticeably higher than in HF condition (Figure 1, Figure 4 and Figure 5).

In the *ein3eil1* mutant, the pericycle proliferation was significantly lower than the wt for IBA treatment (Figure 4), but higher for IAA (Figure 5). Additionally, the treatment with JAMe, in combination with either IBA or IAA, did not cause significant increases in the number of XEs in comparison with each auxin alone (Table 1).

The presence of JAMe in combination with either auxin resulted into changes in the xylary identity.

In fact, both in the wt and in the mutant, each JAMe concentration caused the formation of both protoxylem and metaxylem when combined with IBA (Figure 2M–P, and Table 1). Conversely, treatments with IBA induced only metaxylem in the wt, and only protoxylem in the mutant (Figure 2K-L, and Table 1). In the wt, when 0.01 μM JAMe was combined with IAA, only protoxylem was formed, as for IAA single treatment (Figure 2R,Q in comparison, and Table 1). For treatments with 1 μM JAMe, metaxylem could be detected and became more abundant than protoxylem after treatment with 10 μM JAMe (Figure 2S, and Table 1). Interestingly, in the mutant, where only protoxylem was formed after IAA treatment (Table 1), metaxylem was instead abundantly and increasingly produced, after combined treatments with IAA and increasing concentrations of JAMe (Figure 2T,U, and Table 1).

Collectively, the results showed that JAMe was able to change auxin-induced ectopic xylary cell identity, with an enhancing effect with higher concentrations.

**Figure 6:**
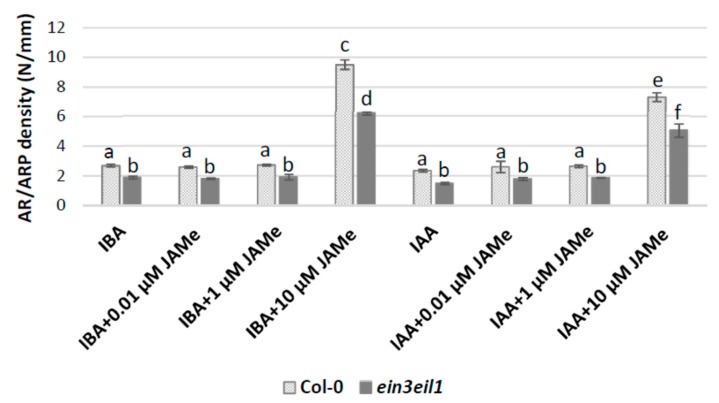
Mean density of adventitious roots (ARs) and adventitious root primordia (ARPs), evaluated as N/mm (± standard error (SE)) in the basal 5 mm of the hypocotyl of Col-0 (wt) and *ein3eil1* double-mutant seedlings cultured with IBA (10 µM) or IAA (10 µM), alone or combined with 0.01, 1 or 10 μM JAMe. Different letters among different treatments within the same genotype, or between the two genotypes within the same treatment, indicate significant differences at least at *p* < 0.05. The same letters indicate no significant difference. Two-way ANOVA followed by Tukey’s post-test. *n* = 30.

As adventitious roots (ARs) and adventitious root primordia (ARPs) are induced by auxin in competition with xylogenesis in the basal hypocotyl of *Arabidopsis* [11], the density of the ARs/ARPs was also evaluated. In the wt, the ARP/AR formation caused by IBA application did not change when also JAMe was present in the medium at 0.01 and 1 μM concentration. Nevertheless, the AR-response increased many folds (*p* < 0.0001) in hypocotyls of seedlings treated with 10 μM JAMe (Figure 6). Similar results were also obtained when IAA was applied in combination with JAMe. However, the AR/ARP production in the combined treatment with IAA and 10 μM JAMe was significantly lower than with 10 μM JAMe + IBA (Figure 6). A similar trend was observed in the mutant treated with the same hormone conditions, even if a significant reduction in AR/ARP density in comparison with the wt was noticed (Figure 6).

### 2.3. Nitric Oxide is an Early Marker of Cell Reactivation in the Hypocotyl Basal Pericycle Involved in Either Xylogenesis or Adventitious Rooting, and its Signal is Enhanced by JAMe

**Figure 7:**
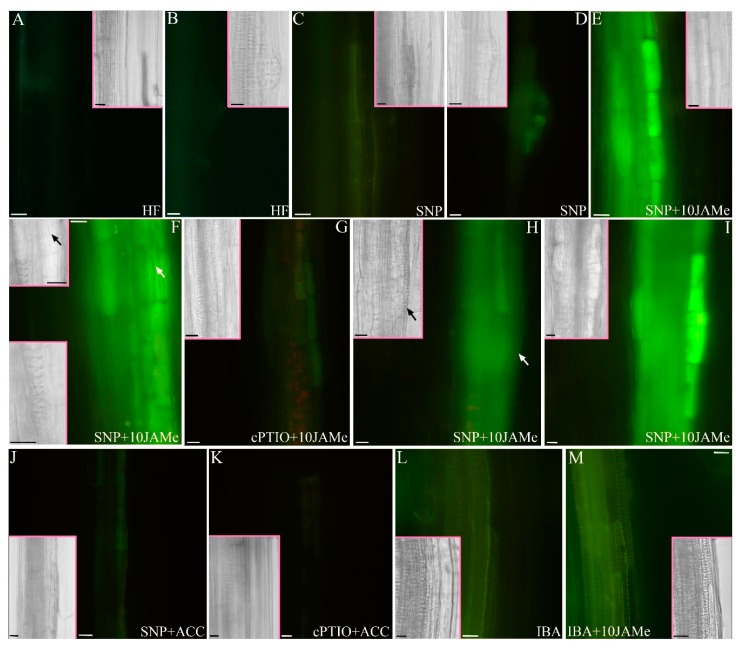
Analysis of NO epifluorescence signal, due to diaminofluorescein-FM diacetate (DAF-FMDA) treatment, in the basal hypocotyl of Col-0 (wt) seedlings dark-grown for 22 DAS. (**A**) Absence of epifluorescence signal in the pericycle periclinal derivatives occasionally formed in hormone-free (HF) condition. (**B**) Absence of epifluorescence signal in an adventitious root primordium derived from anticlinal pericycle divisions in HF condition. (**C**,**D**) Weak signal in correspondence of cells derived from periclinal (**C**) or anticlinal (**D**) divisions of the pericycle in seedlings treated with 50 µM sodium nitroprusside (SNP). (**E**,**F**) Strong NO signal in the pericycle cells (**E**), and their first periclinal derivatives (**F**; arrow in the inset) in 50 µM SNP + 10 µM JAMe (SNP + 10JAMe)–treated seedlings. (**G**) Absence of epifluorescence signal in the pericycle after 100 µM 2-4-carboxyphenyl-4,4,5,5-tetramethylimidazoline-1-oxyl-3-oxide (cPTIO) application to 10 µM JAMe-treated seedlings (cPTIO + 10JAMe). (**H**) Strong NO signal in pericycle periclinal derivatives in the hypocotyl of a seedling treated with SNP + 10JAMe. The signal disappears in the differentiated xylary elements (the arrow indicates an ectopic protoxylem element). (**I**) Strong NO signal in pericycle anticlinal derivatives occasionally formed after SNP + 10JAMe application. (**J**) Weak NO signal in pericycle-derived cells in 50 µM SNP + 0.1 µM ACC (SNP + ACC)-treated seedlings. (**K**) Absence of NO signal in the hypocotyl of seedling cultured with 100 µM cPTIO + 0.1 µM ACC (cPTIO + ACC). (**L**,**M**) Epifluorescence signal in the hypocotyl of seedlings treated with 10 µM IBA (IBA; **L**) or 10 µM IBA + 10 µM JAMe (IBA + 10 JAMe; **M**). The signal intensity increases in the presence of JAMe. All the insets show bright field images of whole-mount seedling hypocotyls. Scale bars = 10 µm (**A**,**B**,**D**–**I**,**K**–**M**, Insets in **A**–**F**,**I**,**K**,**L**), 20 µm (**C**,**J**, Insets in **G**,**H**,**M**), 30 µm (Inset in **J**).

**Figure 8:**
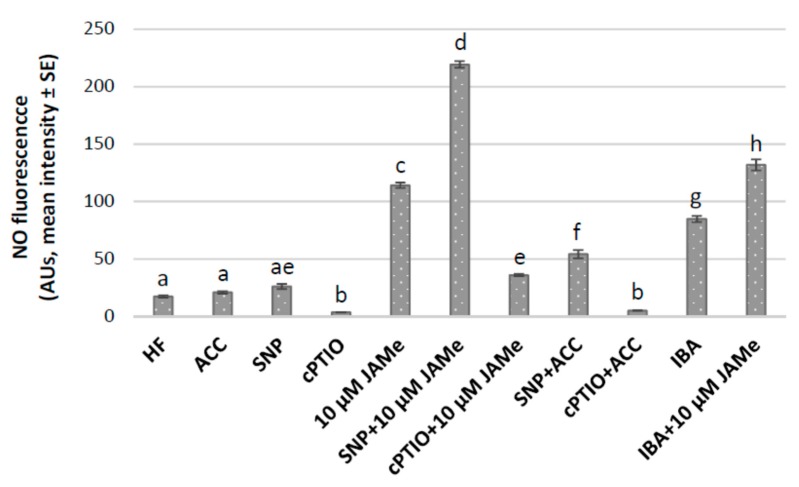
Quantification of the epifluorescence signal due to nitric oxide (NO) production, by means of DAF-FMDA probe, in the basal hypocotyl pericycle cells and in their early derivatives of Col-0 (wt) seedlings grown in darkness for 22 DAS with different treatments. Mean intensity (± standard error (SE)) in Arbitrary Units (AUs) in seedlings cultured in control condition (HF) or with 0.1 µM ACC (ACC), 50 µM SNP (SNP), 100 µM cPTIO (cPTIO), 10 µM JAMe, SNP + 10 µM JAMe, cPTIO + 10 µM JAMe, SNP + ACC, cPTIO + ACC, 10 µM IBA (IBA), 10 µM IBA + 10 µM JAMe. Different letters among treatments indicate significant differences at least at *p* < 0.05. The same letter indicates no significant difference. One-way ANOVA followed by Tukey’s post-test. *n* = 100.

Wild type seedlings cultured in HF conditions or treated with either 10 μM JAMe or 0.1 μM ACC were grown for 22 days in the presence of either the NO donor sodium nitroprusside (SNP) or the NO scavenger 2-4-carboxyphenyl-4,4,5,5-tetramethylimidazoline-1-oxyl-3-oxide (cPTIO). The NO signal was detected with the NO-fluorescent indicator diaminofluorescein-FM diacetate (DAF-FMDA) (Figure 7 and Figure S1), and the derived epifluorescence signal quantified (Figure 8).

The basal hypocotyl of HF-grown seedlings showed a hardly detectable fluorescence signal without addition of SNP in the culture medium. This indicated a very low NO production in the poorly extended periclinal pericycle derivatives, leading to ectopic xylary cells, as well as in the rare anticlinal derivatives and protruding adventitious root primordia (ARPs) (Figure 7A,B, and insets, Figure 8 and Figure S1A). In the presence of SNP, the induced NO production was very low and not significantly different from the HF condition (Figure 7C,D, and insets, and Figure 8). The fluorescence signal was also almost absent in the presence of cPTIO alone (Figure S1B, and Figure 8). In contrast, when the seedlings were cultured with JAMe +/-SNP, an evident NO signal characterized the hypocotyl vasculature, being more intense in pericycle cells (Figure 7E, and inset, Figure 8) and in their first periclinal derivatives (Figure 7F, upper inset and arrows, Figure 8). The signal was instead highly reduced by the cPTIO application in combination with JAMe (Figure 7G and inset, and Figure 8). Moreover, for JAMe +/− SNP treatments, the ability to produce NO decreased with the progress of xylem differentiation becoming almost absent in the differentiated ectopic xylary elements (Figure 7H, inset, and arrows). The ectopic XEs formed were identified as protoxylem type (Figure 7F, lower inset and Figure 7H, inset and arrows), as seen with JAMe treatments in the absence of the NO-donor (Figure 2B, and Table 1). Interestingly, the NO signal was present also in the rarely formed anticlinal derivatives (Figure 7I, and inset), leading to ARP formation, and at the ARP base (Figure S1C), with the NO signal quenching at about ARP VII stage [40] (Figure S1D,E).

In addition, a very low fluorescence signal was observed for the ACC treatment, as measured for HF condition (Figure 8). A weak, but significant, increase appeared instead for ACC treatment combined with SNP (Figure 8). However, the signal intensity in the ACC and SNP combined treatment was much lower than for the 10 μM JAMe and SNP combined treatment (Figure 7E,F,J in comparison and Figure 8), and similarly lowered by cPTIO application (Figure 7K and Figure 8). No changes in xylary cell identity were induced by NO production.

The DAF-FMDA fluorescence was also evaluated in basal hypocotyls of seedlings cultured with JAMe (10 µM) + IBA (10 µM) in comparison with IBA (10 µM) alone, because xylogenesis and AR-formation were more responsive to treatments with IBA than IAA (Figure 6 and Table 1).

The NO production was significantly higher when the seedlings were cultured with IBA and JAMe than only with IBA (Figure 7L,M) or JAMe (Figure 8). This was independent from the initiation of either xylogenesis or rhizogenesis, suggesting a program-independent NO involvement in modulating JAMe effects when combined with IBA.

## 3. Discussion

The data presented show that exogenous JAMe, applied at a specific concentration, induces xylogenesis in *Arabidopsis* seedlings, with a positive involvement of NO and a negative involvement of ET-signalling, and modulates IAA/IBA-determined xylary cell identity.

### 3.1. The Action of JAMe on Xylogenesis is Negatively Affected by ET Signalling by EIN3EIL1

The ectopic formation of protoxylem in the basal hypocotyl is enhanced by JAMe, when applied at 10 μM concentration. Interestingly, JA has been recently demonstrated to induce extra xylem also in the roots of the same ecotype of *Arabidopsis* here investigated, with high increases in xylogenesis with 10 μM JAMe [24], in accordance with the present results for the hypocotyl. Moreover, the JA signalling mutant *coronatine insensitive1-1* (*coi1-1*) does not form ectopic XEs in response to JA, whereas the JA biosynthesis mutant *oxophytodienoate-reductase3* (*opr3*) does form XEs, suggesting that the JA response, instead of synthesis, is responsible for xylogenesis induction [24]. It is, thus, possible that also in the hypocotyl the JA signalling, more than its biosynthesis, is positively involved in the control of xylogenesis. The JASMONATE ZIM-DOMAIN (JAZ) proteins are the target of CORONATINE INSENSITIVE1 (COI1) protein, and COI1–JAZ is the co-receptor of the JA-isoleucine conjugate (JA-Ile), active form of JA, in the responsive target cells, in our case in the hypocotyl pericycle cells. In accordance, *coi1* mutants are JA/JAMe-insensitive ([41] and references therein). It is widely known that the interaction of JA-Ile with COI1 induces the proteolysis of JAZs, releasing MYC2. MYC2 regulates the JA responses by controlling the expression of JA-responsive genes, including, in our hypothesis, those involved in xylogenesis. One of the JA-responsive xylogenic gene might be *ARF17*, belonging to the Auxin Response Factors (ARFs) mediating auxin-induced gene activation [42]. In fact, in *Arabidopsis* TCLs cultured with IBA and Kinetin, JA/JA-Ile is immunolocalized in the xylogenic cells, where *ARF17* is expressed and upregulated by 10 μM JAMe [26]. Moreover, in some reports cytokinin is considered important for xylogenesis to occur [3], but in others it is reported as a negative regulator of xylem development ([24] and references therein). JAMe treatments have been demonstrated to reduce cytokinin effects in the *Arabidopsis* root vasculature, and cytokinin treatments to nullify JAMe promotion of ectopic xylem formation [24]. In accordance, our previous [11] and present results on *Arabidopsis* hypocotyls of dark-grown seedlings show that xylogenesis occurs in the absence of exogenous cytokinin, and that this condition may favour the JA inductive action.

We also show that the *ein3eil1* mutant is JA-sensitive in xylogenesis, whereas it is known to be insensitive in other processes, e.g., the induction of pathogen-related genes and root hair development [39]. Moreover, the xylogenic effect of 10 μM JAMe is observed in the mutant as in the wt, showing that xylogenesis is negatively affected by the interaction of EIN3EIL1 with JA derived by JAMe application. The reduction of the xylary response observed in the wt, after exposition to JAMe combined with the ET-precursor ACC, supports the validity of this interpretation. The JA-sensitivity of *ein3eil1* in xylogenesis is in accordance with the observation that EIN3 and EIL1 are positive regulators only in a specific subset of JA responses [39,43].

### 3.2. Nitric Oxide is a Common Marker of JAMe-induced Xylogenesis and Auxin-Induced Adventitious Rooting Acting Downstream to Pericycle Cell Determination to either Program

Results showed that NO is early produced in the pericycle derivatives, from which either XEs or ARPs are formed, showing that NO is a common marker for both programs. Its presence in pericycle cells, either dividing periclinally to generate XEs or anticlinally to generate ARPs, demonstrates that it is an early marker for either xylogenesis or rhizogenesis. It is known that the exogenous treatment with the NO-donor SNP enhances initiation of root hairs in the root elongation zone through the reorientation of cortical microtubules [44]. Furthermore, cell plate formation is known to be very sensitive to perturbations of the microtubular cytoskeleton, and a peculiar target of nitrotyrosine, coming from NO-mediated post-translational modification ([45] and references therein). It is, thus, possible that a NO-guided rearrangement in the cytoskeleton and cell plate orientation may occur, resulting into either xylogenesis or rhizogenesis. However, NO seems to act downstream on factor(s) causing the pericycle cells to become committed to either program. Interestingly, NO production increases when 10 μM JAMe is applied. NO signalling is involved also in stomatal closure, with NO production highly enhanced by 10 μM JAMe [46]. Previous reports also showed that exogenous JAMe stimulates NO synthase to induce NO production in *Taxus* cells [38], and that JA and NO modulate each other production [47]. In addition, our quantitative data show that NO production, either with ACC single treatment, or when ACC was combined with SNP, was much lower than with 10 μM JAMe, combined or not with the same NO-donor compound. This suggests an antagonistic connection between JAs and ET in the control of xylogenesis, related to NO levels and, possibly, NO signal activity.

Interestingly, the disappearance of the NO signal occurs only later in xylogenesis, i.e., when the XEs become mature, while in rhizogenesis it occurs at an early stage (stage VII) of ARP formation. This means that a sustained NO production is needed until PCD and lignin deposition are concluded. On the other hand, the disappearance of the NO signal occurs in a still widely meristematic condition during rhizogenesis. In accordance, in *Zinnia elegans* a NO burst occurs before the processes of secondary cell wall formation and cell autolysis, in both differentiating xylem of vascular bundles and trans-differentiating xylary cells of leaf mesophyll ([29] and references therein). Moreover, in accordance with our results, in *Populus* roots, NO signalling contributes to the onset of xylary differentiation and to further stages of maturation, but it is not observed in the mature vessels [34].

It is known that IBA induces AR-formation by conversion into IAA, and this involves NO activity ([35] and references therein). Present and past results together show that NO is present in the ARPs meristematic domes in TCLs [35] and in planta (present results). It is possible that NO signalling is a long-lasting process in xylogenesis, in comparison with rhizogenesis, because it is necessary for consecutive events until xylary maturation. Conversely, ARPs do not need prolonged NO activity, being capable to sustain their growth after quiescent centre definition in their meristematic dome. This normally occurs at stage VII of ARP development [7,40], i.e., when the NO signal is here shown to be quenched.

Moreover, the DAF-FMDA fluorescence revealed that NO production was higher in the presence of JAMe (10 µM) combined with IBA (10 µM), than with IBA (10 µM) alone, independently of the initiation of either xylogenesis or rhizogenesis. This strengthens the idea of a program-independent NO involvement in both processes, as well as the concurrent role of JAMe combined with IBA in enhancing early NO formation.

Auxin (natural or synthetic) is necessary to trigger xylogenesis ([1] and present results), and IAA and its precursor IBA are present in the hypocotyls of dark-grown *Arabidopsis* seedlings [19]. Moreover, it is known that endogenous auxin accumulates before the formation of XEs in numerous plants/culture systems, and this occurs both after either a dedifferentiation in the target cells or their direct trans-differentiation ([11] and references therein). In particular, in *Arabidopsis* hypocotyls endogenous auxin accumulates in the basal pericycle before the first divisions occur [40]. Present data show that only a rare xylary formation is induced by the endogenous hormonal input (HF condition), whereas an enhancement is detected in the presence of 10 µM JAMe. Although a synergism of auxin and NO during the activation of cytokinesis has been reported [48], it still remains to be explained how the endogenous auxin with exogenous JAMe direct the basal pericycle cells towards the realization of the xylogenic program and not root formation, by using the same NO signalling molecule.

Surprisingly, xylogenesis does not increase with any JAMe concentration combined with either IBA or IAA, in comparison with the auxins single treatments. Additionally, the IBA/IAA-induced adventitious rooting is enhanced by the same JAMe concentration (10 μM) that enhances xylogenesis when applied alone. This suggests that JA acts as a common player between the two programs in the same target cells, with a possible different action depending on the endogenous auxin levels. Xylogenesis might need lower levels of auxin to be initiated, in accordance with the endogenous IAA levels monitored in the absence/presence of IBA in *Arabidopsis* dark-grown seedlings [19]. Moreover, this possibility has been also previously hypothesized based on the activity of the auxin influx carrier AUX1 in switching between the two developmental programmes [7]. In addition, it has been suggested that the JA-mediated activation of auxin response in *Arabidopsis* roots requires the function of AUX1, and JAMe upregulates AUX1 expression [49].

### 3.3. The Auxin-Determined Xylary Cell Identity is Modulated by JAMe

It is known that auxins are essential for determining xylem cell identity, with the formation of auxin maxima in the target cells mediating the process [50]. In various plant species, VASCULAR-RELATED NAC-DOMAIN6 and 7 (VND6 and 7) are master TFs in the control of XE identity, because *VND6* and *VND7* ectopic expression causes the trans-differentiation of either metaxylem or protoxylem elements ([51] and references therein). In fact, in *Arabidopsis* and poplar roots, these genes have been proposed as transcription switches for metaxylem and protoxylem formation [52]. VND7, in particular, exhibits a critical role in regulating protoxylem formation in the root, and metaxylem formation in both root and shoot [53]. In addition, SCARECROW (SCR) TF is induced by auxin [54], and forms with SHORT ROOT (SHR) TF a complex that produces the microRNA 165/166, involved in the destabilization of *HD-ZIP III* genes [55], determinant for metaxylem specification [7]. Both *Arabidopsis shr* and *scr* null mutants produce ectopic metaxylem in dark-grown seedlings [7], supporting a possible involvement of SHR-SCR complex activities in the specific realization of metaxylem. Moreover, it has been recently demonstrated that the metaxylem and protoxylem identity might be mutually repressed. Glycogen Synthase Kinase 3 (GSK3) proteins, like BRASSINOSTEROID INSENSITIVE 2 (BIN2), BIN2-LIKE 1 (BIL1), BIL2, SHAGGY-RELATED KINASE 11 (ATSK11) and ATSK13 have been demonstrated to interact with PHLOEM INTERCALATED WITH XYLEM (PXY), with this interaction seeming to specify repression of xylem identity [56].

The present results show that JAMe changes auxin-induced ectopic xylary cell identity, with an enhancing effect caused by the increase in its concentration. This is the first report of a role for JAs in changing xylary cell identity.

It is known, and here confirmed, that ectopic XE formation is stimulated by the exogenous application of IAA and IBA at the same concentration (10 μM) in dark-grown *Arabidopsis* hypocotyls. Nevertheless, the two auxins affect differently the xylary identity, with metaxylem induced by IBA and protoxylem by IAA [11 and present results].

We showed that the pericycle periclinal proliferation with xylogenesis in the wt was comparable for each auxin and JAMe (10 μM) individual treatments, and higher than HF or ACC, confirming the xylogenic role of the two exogenously applied auxins, but also of JAMe acting in combination with the endogenous hormonal pool. However, the presence of JAMe in combination with either IBA or IAA did not increase the number of the XEs formed in comparison with IBA/IAA alone, but affected xylary identity.

Independently of treatment and genotype, we observed only ectopic protoxylem formation after JAMe treatment, as observed for HF condition, thus showing that the applied JAMe did not change the xylary cell identity induced by the endogenous hormones. This was also demonstrated in the mutant impaired in ET-perception, but JA-sensitive, excluding a possible involvement of the EIN3EIL1 complex in this process. However, in the wt, JAMe, especially at the highest concentration, induced both ectopic protoxylem and metaxylem when combined with either IBA or IAA. Interestingly, in the mutant, forming only protoxylem after either IAA or IBA single treatments, metaxylem was instead abundantly formed when JAMe was applied, especially at 10 µM, combined with each auxin. Altogether, the results show that JAMe-modulation of ectopic xylem identity induced by exogenous IAA or IBA bypassed ET-perception by EIN3EIL1.

**Figure 9:**
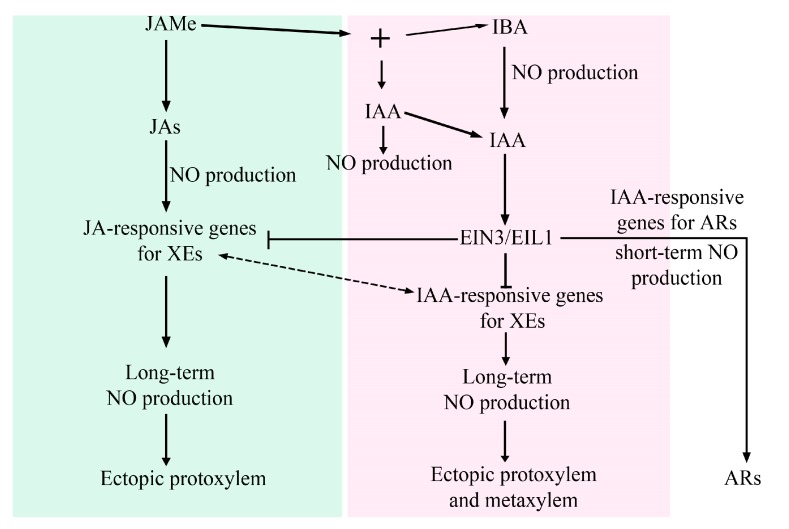
Proposed model for jasmonates (JAs) and auxin (IAA/IBA) roles in the formation of ectopic xylary cells (XEs) in the pericycle derivative cells of *Arabidopsis* basal hypocotyl of seedlings cultured with JAMe (10 µM) +/− IBA (10 µM) or IAA (10 µM). The pathway leading to the formation of adventitious roots (ARs) as an alternative to XE formation is also shown. The green part of the figure shows JA’s effect in the absence of exogenous auxin. JAs derived by JAMe demethylation [23] induce NO production. The NO signal triggers the expression of JA-induced genes responsible for XE formation. This occurs through a negative interaction with ET-signalling by EIN3/EIL1. In the following steps of the differentiation process, a long-term NO production occurs and lasts until the maturation of ectopic protoxylem elements. When IBA is applied with JAMe (the pink part of the figure) is converted into IAA, also inducing NO-formation [35]. When IAA is alternatively applied with JAMe, NO-formation may also occur [27]. Cell perception of the endogenous IAA derived by the application of the two exogenous auxins is associated with a positive crosstalk with ET through EIN3EIL1 in AR-formation [11], and with a negative crosstalk in xylogenesis (present results). Different pathways of NO production occur in the two programs. A short-term NO production is associated with the expression of the IAA-induced genes involved in AR-formation, whereas a long-term NO production is proposed to be induced after the possible interaction of JA-responsive and IAA-responsive genes for XE-formation thereby leading to the ectopic formation of both protoxylem and metaxylem.

In conclusion, the basic findings of this research add new elements to the intricate switching of xylogenesis and adventitious rooting in the same cells. Here, JAs are demonstrated to be actors in both developmental programs, and in the modulation of xylary cell identity, with an important mediation by NO, as summarized in the model of Figure 9.

The present findings may be also exploited to implement practical aspects related to the creation of plants with improved xylem, favouring plant survival under water stress conditions, or for biotechnological purposes, e.g., for the optimization of biofuels and biomaterials production.

## 4. Materials and Methods

### 4.1. Plant Growth

One hundred seeds of Col-0 ecotype of *Arabidopsis thaliana* (L.) Heynh and of its homozygous double mutant *ein3eil1* [39] (provided by Hongwei Guo, Peking University, China) were sown, after sterilization, on square Petri plates (12 cm × 12 cm; 10 seeds per plate) containing full strength Murashige and Skoog (MS) [57] salts supplemented with 0.55 mM myo-inositol (Fluka, Buchs, Switzerland), 0.1 µM thiamine-HCl (Sigma-Aldrich, St. Louis, MO, USA), 1% (*w*/*v*) sucrose (Sigma-Aldrich) and 0.8% (*w*/*v*) agar (Sigma-Aldrich) (pH 5.7). As an alternative to this Hormone Free (HF) medium, either JAMe (0.01, 1 or 10 µM) (Duchefa Biochemie B.V, Haarlem, NH, USA), or ACC (0.1 µM) (Sigma-Aldrich), alone or combined with 10 µM JAMe, were added. The final JAMe concentrations in the media were obtained taking the exact amounts from a stock solution (10^−3^ M) in 10% ethanol. To dissolve the JAMe, diluted ethanol was preferred to pure ethanol, suggested by the producer (Duchefa Biochemie B.V, Haarlem, NH), in order to keep the final ethanol concentration in the media below 0.1% to avoid possible negative effects on germination [58].

Media containing the components of the HF medium and either IAA or IBA (both from Sigma-Aldrich), at 10 µM concentration, alone or combined with JAMe (0.01, 1 or 10 µM), were also prepared. Moreover, treatments with either 50 µM of the NO donor sodium nitroprusside (SNP; Merck, Darmstadt, Germany) or 100 µM of the NO scavenger 2-4-carboxyphenyl-4,4,5,5-tetramethylimidazoline-1-oxyl-3-oxide (cPTIO; Sigma-Aldrich) were performed either in HF condition or with JAMe (10 µM) or ACC (0.1 µM). The media were sterilized by autoclaving at 120 °C for 20 min. IBA and IAA were added before autoclaving taking the appropriate volume from stock-solutions (10^−3^ M). Sterile stock-solutions of JAMe (10^−3^ M), ACC (10^−3^ M), SNP (10^−2^ M) and cPTIO (10^−2^ M) were prepared by filtering (with a 0.22 µm pore filter), and the appropriate volume was taken to reach the final concentration in the already autoclaved medium, after the medium temperature decreased up to 45–50 °C.

Independently from the treatment, after sowing, the seeds were stratified for three days at 4 °C under continuous darkness and exposed to white light (intensity 100 µE·m^−2^·s ^−1^) for about 6 h, to induce germination. The plates were then placed in vertical position under continuous darkness until 22 days after stratification (DAS), at 22 ± 2 °C.

### 4.2. Histological Analysis

After 22 DAS, 30 seedlings per genotype and per treatment were fixed, dehydrated, embedded in Technovit 7100 (Heraeus Kulzer, Germany), longitudinally sectioned (8 µm thickness) with the Microm HM 350 SV microtome (Microm, Walldorf, Germany), and stained with 0.05% toluidine blue (all procedures according to [40]). Sections were taken from the basal portion (5 mm in length) of the hypocotyl, according to Fattorini and co-workers [11], and observed with a Leica DMRB microscope; images were acquired with a DC500 camera (Leica, Wetzlar, Germany).

### 4.3. Nitric Oxide Detection

Intracellular NO content in 22d old Col-0 seedlings, cultured either in HF medium or treated with 10 µM JAMe, or 0.1 µM ACC, with/without SNP (50 µM) or cPTIO (100 µM), or with 10 µM IBA or 10 µM IBA + 10 µM JAMe, was monitored using the cell-permeable diacetate derivative diaminofluorescein-FM (DAF-FMDA; Sigma). Seedlings were incubated in 20 mM HEPES/NaOH buffer (pH 7.4) supplemented with 5 μM DAF-FMDA for 20 min [59] at 22 DAS, after having verified that no significant epifluorescence signal was detectable with the buffer alone (Figure S2). The excess of the fluorescent probe was removed by washing the seedlings for three times with the buffer, after which they were observed using a Leica DMRB microscope equipped with the specific set of filters (EX 450–490, DM 510, LP 515). The images were acquired with a Leica DC500 digital camera and analyzed with the IM1000 image-analysis software (Leica). The intensity of the green fluorescent signal was quantified in the basal hypocotyl of ten seedlings per treatment. Ten measures per seedling were randomly carried out in the pericycle cell derivatives using the ImageJ software (National Institute of Health, Bellevue, WA, USA), and expressed in arbitrary units (AUs; from 0 to 255). The values were averaged and normalized to those measured in hypocotyls incubated in the buffer without the fluorescent probe according to Fattorini and coworkers [35].

### 4.4. Measurement Procedures and Statistical Analysis

The hypocotyls of 30 seedlings per genotype and treatment were cut into three portions. Measures of the radial extension of the vascular system, including the de novo formed cells by the pericycle periclinal divisions, were carried at the middle of the hypocotyl basal portion, according to Fattorini and co-workers [11], and expressed as mean values (± standard error (SE)) In the same hypocotyl portion, the ectopic XEs present in an area of 150 × 150 µm^2^ were counted and quantified as mean numbers (±SE). The protoxylem vs. metaxylem identity was determined and expressed as percentage on the total of the de novo formed XEs. Adventitious roots were counted in the same hypocotyl portions and evaluated as mean density (±SE).

One-way or two-way analysis of variance (ANOVA, *p* < 0.05) was used to compare the effects of different treatments or different treatments and genotypes, respectively, and, if ANOVA showed significant effects, Tukey’s post-test was applied (GraphPad Prism 6.0, GraphPad Software, Inc., La Jolla, CA, USA). All the experiments were repeated three times, with very similar results.

## Figures and Tables

**Table 1:** Quantification of ectopic xylary elements (XEs) detected in the basal hypocotyl of Col-0 and *ein3eil1* seedlings. Mean number (± standard error (SE)) of XEs and percentage of ectopic protoxylem and metaxylem elements detected in an area of 150 × 150 µm^2^ in the middle portion of the basal hypocotyl with different treatments. ACC, IBA and IAA were always used at 0.1 µM, 10 µM and 10 µM, respectively. Different letters among values within the same column indicate significant differences at least at *p* < 0.05. The same letter indicates no statistical difference. One-way ANOVA followed by Tukey’s post-test. *n* = 30.

Treatment	Col-0 Ectopic XEs Mean Number (± SE)	*ein3eil1* Ectopic XEs Mean Number (± SE)	Col-0 Ectopic Protoxylem %	Col-0 Ectopic Metaxylem %	*ein3eil1* Ectopic Protoxylem %	*ein3eil1* Ectopic Metaxylem %
**HF (control)**	0.5 ± 0.2 ^a^	0.6 ± 0.3 ^a^	100	0	100	0
**0.01 µM JAMe**	1.3 ± 0.3 ^a^	2.3 ± 0.7 ^b^	100	0	100	0
**1 µM JAMe**	1.0 ± 0.3 ^a^	2.4 ± 0.7 ^b^	100	0	100	0
**10 µM JAMe**	2.8 ± 0.3 ^b^	2.5 ± 0.4 ^b^	100	0	100	0
**0.1 µM ACC**	1.1 ± 0.3 ^a^	0.6 ± 0.2 ^a^	45.5	54.5	100	0
**10 µM JAMe + ACC**	0.8 ± 0.2 ^a^	2.4 ± 0.3 ^b^	100	0	100	0
**10 µM IBA**	4.4 ± 0.6 ^b^	2.0 ± 0.1 ^b^	0	100	100	0
**IBA + 0.01 µM JAMe**	3.5 ± 0.4 ^b^	2.4 ± 0.3 ^b^	17.6	82.4	20.7	79.3
**IBA + 1 µM JAMe**	3.7 ± 0.8 ^b^	2.3 ± 0.4 ^b^	22.7	77.3	22.2	77.8
**IBA + 10 µM JAMe**	4.0 ± 0.6 ^b^	3.6 ± 0.2 ^b^	61.9	38.1	50	50
**10 µM IAA**	2.8 ± 0.5 ^b^	2.0 ± 0.4 ^b^	100	0	100	0
**IAA + 0.01 µM JAMe**	2.5 ± 0.4 ^b^	1.8 ± 0.4 ^b^	100	0	35	65
**IAA + 1 µM JAMe**	2.5 ± 0.2 ^b^	2.2 ± 0.3 ^b^	33.3	66.7	25	75
**IAA + 10 µM JAMe**	2.6 ± 0.5 ^b^	3.8 ± 0.8 ^b^	7.7	92.3	9	91

## Supplementary Materials

Supplementary materials can be found at https://www.mdpi.com/1422-0067/20/18/4469/s1.

## Abbreviations

ACC	1-Aminocyclopropane-1-carboxylic acid
ANOVA	Analysis of variance
AR	Adventitious root
ARP	Adventitious root primordium
AU	Arbitrary unit
cPTIO	2-4-carboxyphenyl-4,4,5,5-tetramethylimidazoline-1-oxyl-3-oxide
DAF-FMDA	Diaminofluorescein-FM diacetate
DAS	Days after stratification
ET	Ethylene
HF	Hormone-free
IAA	Indole-3-acetic acid
IBA	Indole-3-butyric acid
JA	Jasmonic acid
JAMe	Jasmonic acid methyl ester
SE	Standard error
SNP	Sodium nitroprusside
TF	Transcription factor
XE	Xylary element
