# Supplementary material for: Jasmonic Acid Methyl Ester Induces Xylogenesis and Modulates Auxin-Induced Xylary Cell Identity with NO Involvement
Source: Int J Mol Sci. 2019 Sep 10;20(18):4469. doi: 10.3390/ijms20184469 (PMC6770339; doi:10.3390/ijms20184469)
Supplement: Supplementary file 1 [file ijms-20-04469-s001.pdf]

Supplementary Materials

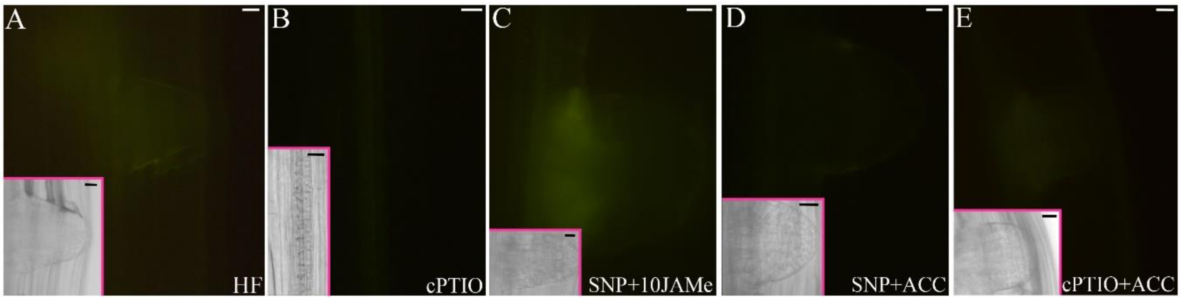

Figure S1. Nitric oxide (NO) epifluorescence signal at the basal hypocotyl of Col-0 seedlings grown in darkness for 22 DAS. (**A**) Basal hypocotyl of HF-treated Col-0 seedlings showing no fluorescence signal by DAF-FMDA in a protruding adventitious root primordium (ARP) (visible in the bright field inset). (**B**) No fluorescence signal detectable in the ectopically forming protoxylem (visible in the bright field inset) in the presence of cPTIO (100 μM). (**C**) NO fluorescence signal by DAF-FMDA at the base of a protruding ARP (visible in the bright field inset) formed in the basal hypocotyl of a 10 μM JAMe plus SNP (50 μM) (SNP+10JAMe)-cultured seedling. (**D-E**) Absence of NO-epifluorescence signal in ARPs at stage VII of development formed in the basal hypocotyl of seedlings cultured with ACC (0.1 μM) plus either SNP (50 μM) (D) or cPTIO (E). Insets show the same ARPs in bright field. Scale bars = 10 μm (**B**, **D**, Inset in **B**), 20 μm (**A**, **C**, **E**, Insets in **A**, **C**-**E**).

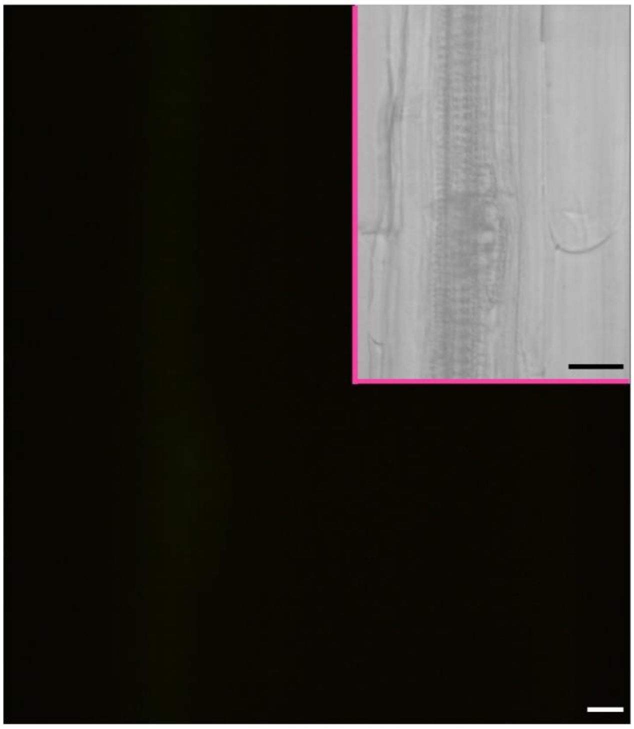

Figure S2. Absence of NO epifluorescence signal in the absence of the fluorescent probe (DAF-FMDA). Epifluorescence and bright field images (inset) of the basal hypocotyl of a Col-0 seedling incubated in 20 mM HEPES/NaOH buffer (pH 7.4) where no epifluorescence signal is detectable. Scale bars = 10 μm, 20 μm in Inset.
